# TAZ induces lung cancer stem cell properties and tumorigenesis by up-regulating ALDH1A1

**DOI:** 10.18632/oncotarget.16430

**Published:** 2017-03-21

**Authors:** Jihang Yu, Adel Alharbi, Hongchao Shan, Yawei Hao, Brooke Snetsinger, Michael J. Rauh, Xiaolong Yang

**Affiliations:** ^1^ Department of Pathology and Molecular Medicine, Queen's University, Kingston, Ontario, Canada; ^2^ Department of Laboratory Medicine, Umm Al-Qura University, Makkah, Makkah Province, Saudi Arabia

**Keywords:** Hippo, TAZ, ALDH1A1, lung cancer, cancer stem cells

## Abstract

Recent studies suggest that lung cancer stem cells (CSCs) may play major roles in lung cancer. Therefore, identification of lung CSC drivers may provide promising targets for lung cancer. TAZ is a transcriptional co-activator and key downstream effector of the Hippo pathway, which plays critical roles in various biological processes. TAZ has been shown to be overexpressed in lung cancer and involved in tumorigenicity of lung epithelial cells. However, whether TAZ is a driver for lung CSCs and tumor formation *in vivo* is unknown. In addition, the molecular mechanism underlying TAZ-induced lung tumorigenesis remains to be determined. In this study, we provided evidence that constitutively active TAZ (TAZ-S89A) is a driver for lung tumorigenesis *in vivo* in mice and formation of lung CSC. Further RNA-seq and qRT-PCR analysis identified *Aldh1a1*, a well-established CSC marker, as critical TAZ downstream target and showed that TAZ induces *Aldh1a1* transcription by activating its promoter activity through interaction with the transcription factor TEAD. Most significantly, inhibition of ALDH1A1 with its inhibitor A37 or CRISPR gene knockout in lung cancer cells suppressed lung tumorigenic and CSC phenotypes *in vitro*, and tumor formation in mice *in vivo*. In conclusion, this study identified TAZ as a novel inducer of lung CSCs and the first transcriptional activator of the stem cell marker ALDH1A1. Most significantly, we identified ALDH1A1 as a critical meditator of TAZ-induced tumorigenic and CSC phenotypes in lung cancer. Our studies provided preclinical data for targeting of TAZ-TEAD-ALDH1A1 signaling to inhibit CSC-induced lung tumorigenesis in the future.

## INTRODUCTION

Lung cancer is the most common cancer worldwide with a 5-year survival rate of < 15% due to lack of druggable targets, development of drug resistance to therapy, or metastasis [1, 2]. Therefore, there is an urgent need to identify new therapeutic drug targets for successful treatment. Mounting evidence suggest that lung tumorigenesis is mostly driven by CSC or tumor initiating cells (TICs) that are more aggressive (metastatic) and refractory to conventional chemotherapeutics [3]. This explains why most current therapies fail to significantly extend lung cancer patients' life span, although they can transiently suppress tumor growth at the beginning of the treatment. Therefore, identification of genes responsible for the development and maintenance of lung CSC phenotypes is critical for the development of drugs specifically targeting CSCs for successful lung cancer therapy.

The transcriptional co-activator with PDZ-binding domain (TAZ) or WWTR1 is a WW domain-containing transcriptional co-activator and downstream component of the tumor suppressor Hippo pathway, which plays critical roles in organ size control, stem cell self-renewal, tumorigenesis, metastasis, and drug resistance [4–7]. Recently, we and others have identified TAZ as a novel oncogene that is overexpressed in non-small cell lung cancer (NSCLC) cells and patients [8, 9]. We have shown that over-expression of TAZ causes increased cell proliferation and transformation, whereas knockdown of TAZ in NSCLC cells inhibit their tumorigenic phenotypes [8]. In addition, it has also been shown that high levels of TAZ contribute to metastatic lung cancer and are correlated with poor patient survival [10]. Together, these studies clearly demonstrate that TAZ is causally linked to lung cancer development and progression, therefore, it may be a promising therapeutic target for lung cancer. However, although TAZ was previously shown to be a regulator of CSC in other cancers such as breast cancer [11–14], whether it plays any role in lung CSC and how TAZ induces lung tumorigenesis remain largely unknown.

The ALDH (Aldehyde dehydrogenase) family is a group of cytosolic isoenzymes that catalyze the oxidation of aldehydes and retinol in cells and play important roles in cellular detoxification and controlling metabolism of retinoic acid (RA) crucial for normal growth, differentiation, and development of adult organs and tissues in vertebrates [15]. Recently, Aldh1a1, a key member of ALDH family, has been shown to be a marker for CSCs in many types of cancers including lung cancer [15, 16]. ALDH1a1 is up-regulated in lung CSC and its expression is positively correlated with the stage and grade of lung cancer patients and related to a poor prognosis [16]. However, the molecular mechanism involved in the up-regulation of Aldh1a1 in CSC remains unclear.

In this study, we have provided strong *in vitro* and *in vivo* evidence that TAZ can induce lung CSC phenotypes and tumorigenesis through TEAD-dependent transcriptional up-regulation of Aldh1a1.

## RESULTS

### Establishment of an *in vivo* TAZ-overexpressing xenograft mouse model

**Figure 1:**
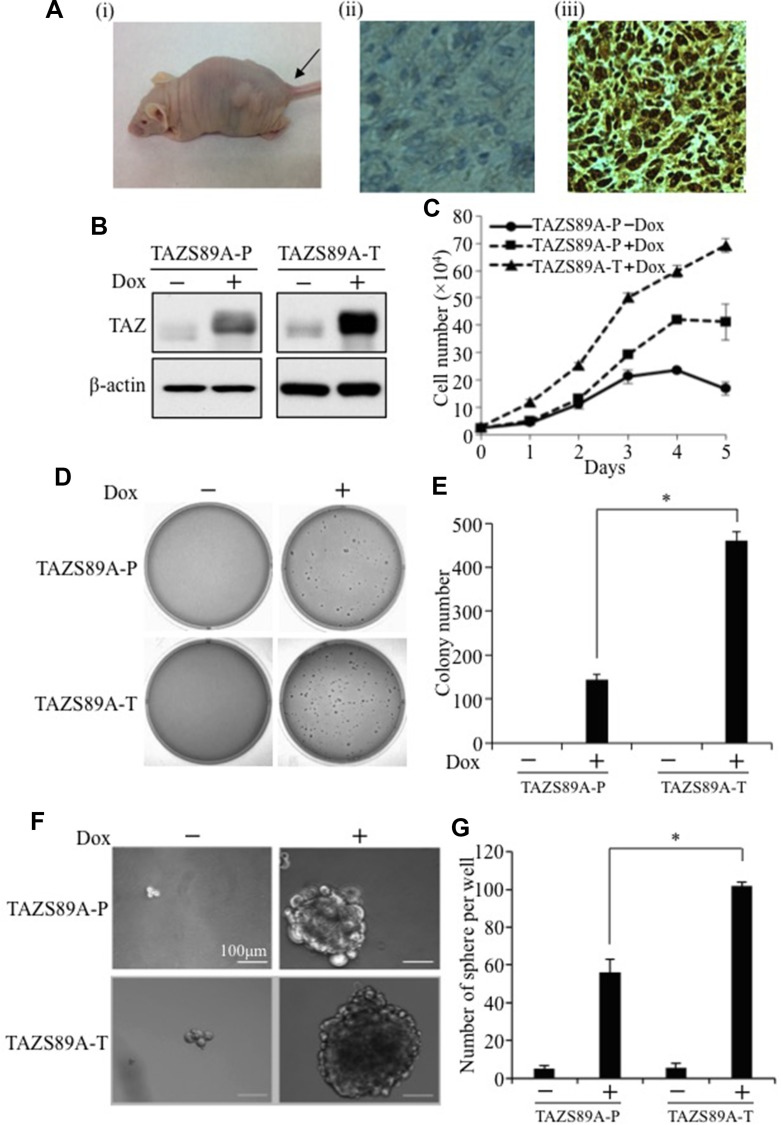
Establishment of an *in vivo* xenograft TAZ-overexpressing mouse model (**A**) Overexpression of TAZ-S89A in mouse immortalized lung epithelial cells (E10) caused highly malignant NSCLC tumor formation. Tumorigenesis assay was performed by subcutaneously injecting about 3 × 10^6^ E10-TAZ-S89A cells into two-sides of nude mice. E10-TAZ-S89A cells caused large tumors (i) in two weeks. Two week later, the tumors were fixed, sectioned, and subjected to H&E staining and IHC. H&E staining with antibody incubation of E10-TAZ-S89A tumor section showed high-grade, poorly-differentiated carcinoma (ii). IHC staining for TAZ expression using TAZ antibody (1:300 dilution, BD Biosciences) showed that TAZ was overexpressed in the nuclei (iii). Pictures were taken using TE200 Nikon Inverted Fluorescent Microscope (Nikon, Montreal, Canada) as 20× magnification. (**B**) Western blot analysis of TAZ-S89A expression. Ten μg of cell lysate extracted from E10-TAZS89A-P or E10-TAZ-S89A-T cells in the absence (−) or presence (+) of Dox were subjected to WB analysis using anti-TAZ (1:1000, BD Biosciences) and anti β-actin (1:10,000 Sigma, Oakville, Canada) antibodies. β-actin was used as an internal loading control. (**C**) Cell proliferation assay. Triplicates of 1.5 × 10^4^ E10-TAZ-S89A-P or tumorigenic E10-TAZ-S89A-T cells were seeded into each well of 12-well plates, then untreated (−) or treated (+) with Dox. Cell numbers were counted on days 1, 2, 3, 4, 5, and 6 after plating. The experiments were repeated at least three times. Data are shown as means ± S.D. “*” represent significant difference. (**D**–**E**) Soft agar assay. Triplicates of 2×10^3^ TAZ-S89A-P or E10-TAZ-S89A-T cells were mixed with 0.4% agarose in growth media and overlaid on 0.8% agarose in 6-well plates, followed by incubation in the absence or presence of Dox (2 μg/ml). Colony formation was examined after culturing for 18 days (D). Colony numbers were counted by Bio-Rad Gel Doc System (Bio-Rad, Mississauga, Canada). Data are shown as means ± S.D. “*” represent significant difference (*P* < 0.05) in *t*-test (E). (**F**–**G**) Sphere formation assay. Triplicates of 1 × 10^4^ cells were seeded into each well of ultra-low-attachment 6-well plates, followed by incubation in the absence or presence of Dox (1 μg/ml). Spheres were set as larger than 100μm. Sphere size are shown as pictures taken as 20× magnification (F). The data are shown as means ±S.D. (*n* = 3). “*” represent significant difference (*P* < 0.05) in *t*-test (G).

TAZ has been identified as a novel oncogene that is overexpressed in NSCLC cell lines, and knockdown of TAZ by shRNA in NSCLC cell lines inhibits cell proliferation, transformation and tumorigenesis [8]. In order to mimic TAZ overexpression in NSCLC, a TAZ gain-of-function model was established by overexpression of TAZ in a TAZ-low human immortalized non-tumorigenic lung epithelial cell line (HBE135). Surprisingly, overexpression of human TAZ in HBE135 cells increased cell proliferation and caused cell transformation but did not cause tumor formation *in vivo* in nude mice [8]. Here, we overexpressed the constitutively active form of TAZ (TAZ-S89A), which has superior oncogenic effects to wild-type TAZ due to mutation of its upstream kinase and suppressor LATS phosphorylation site, in both HBE135 and E10 mouse non-tumorigenic lung epithelial cells using a lentiviral Dox-inducible system. HBE135-TAZ-S89A and E10-TAZ-S89A cells were subcutaneously injected into nude mice, followed by Dox treatment. Remarkably, in the presence of Dox, E10-TAZ-S89A formed large-size tumor in two weeks, whereas HBE135-TAZ-S89A formed tiny tumor after 2 months. Therefore, we used cell line derived from tumor caused by E10-TAZ-S89A in our further experiments. Hematoxylin and eosin (H&E) staining and immunohistochemical (IHC) analysis of tumor histology and TAZ expression, respectively showed that overexpression of TAZ-S89A in E10 lung epithelial cells stimulates tumor formation characterized by high-grade poorly differentiated carcinoma with high nuclear (activated) TAZ expression (Figure 1A). Formation of such highly malignant tumors after TAZ-S89A induction in two weeks confirms that TAZ is indeed a driver of tumorigenicity in lung cancer. To further explore the molecular mechanism underlying TAZ-S89A-induced tumorigenesis, we isolated E10-TAZ-S89A cells from tumor xenografts (E10-TAZ-S89A-T). The establishment of the new tumor-derived cell line was confirmed by detecting TAZ-S89A expression by Western blot (WB) (Figure 1B). Compared to parental E10-TAZ-S89A (TAZ-S89A-P), E10-TAZ-S89A-T cells have significant increase in TAZ expression (Figure 1B), cell proliferation (Figure 1C) and transformation (Figure 1D and 1E). Most significantly, they obtained higher cancer stem cell phenotypes with increased sphere size (Figure 1F) and number (Figure 1G) as demonstrated by sphere formation assay, suggesting that the new-tumor-derived cells have high percentage of CSC and tumorigenic activity.

### Functional domains mediating TAZ-induced tumorigenesis and cancer stem cell phenotype

**Figure 2:**
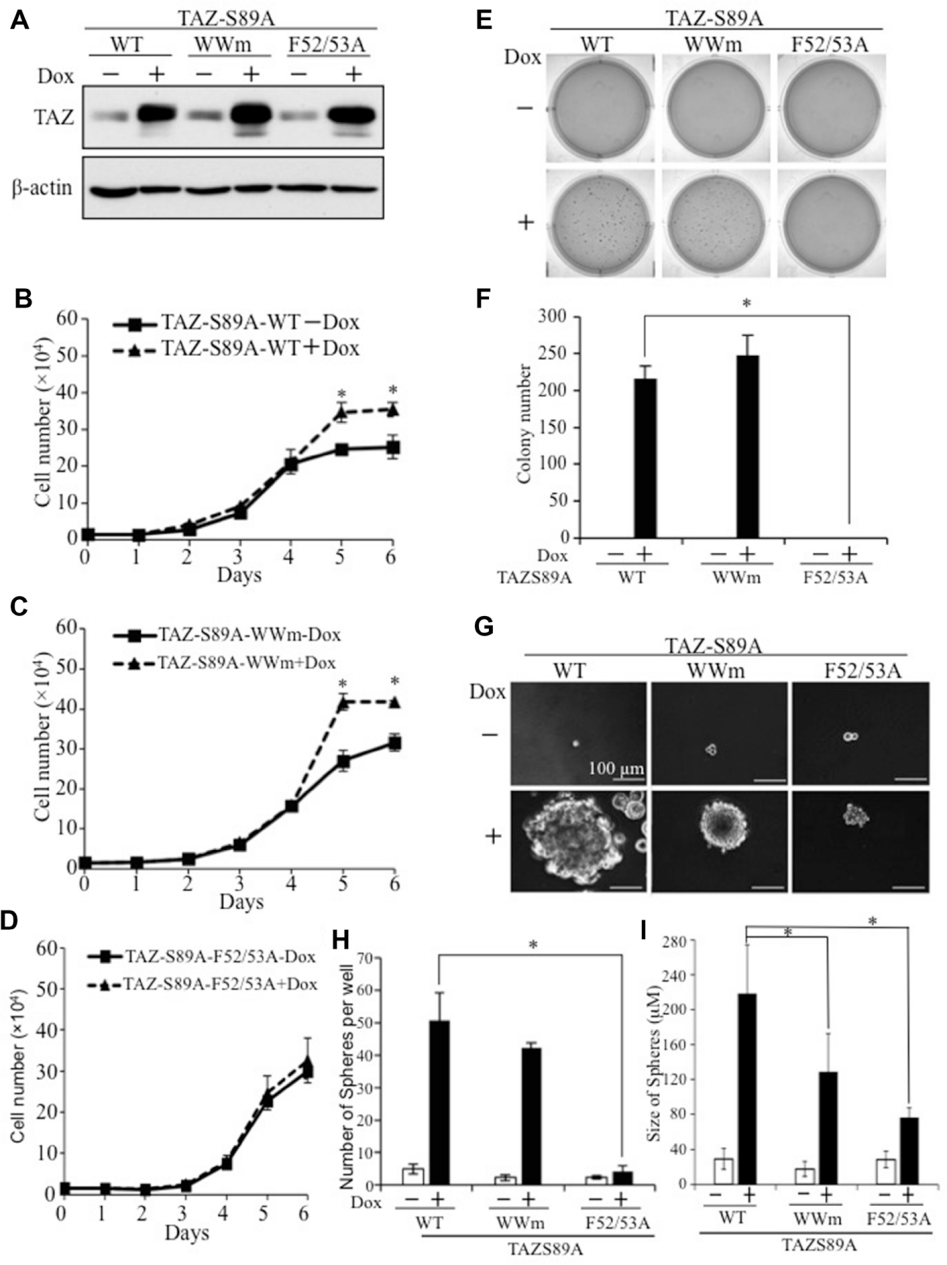
Identification of functional domains mediating TAZ-induced tumorigenic and cancer stem cell phenotypes (**A**) Western blot analysis of TAZ-S89A expression. E10 cells expressing WT, WWm, or E52/53A TAZ-S89A were incubated in the absence (−) or presence (+) of Dox for 2d, followed by protein extraction and WB analysis. (**B**–**D**) Cell proliferation assays. E10-TAZ-S89A wild-type (WT) (B), WW mutant (WWm) (C), and TEAD-binding domain mutant (F52/53A) (D) cells were incubated in the absesnce (−) or presence (+) of Dox for 1–6 days. Experimental procedures were as described in Figure 1C. (**E**, **F**) Soft-agar assays. Procedure and data analysis were as described in Figure 1D–1E. (**G**–**I**) Sphere formation assays. Experimental procedures and data analysis were as described in Figure 1F–1H. Averaged size (diameter) of spheres were also presentated (I).

Although interaction with TEAD has been shown to be critical for TAZ-induced tumorigenesis, conflicting results have been reported on the roles of the WW domain of TAZ in its function [14, 17–19]. To further understand the molecular mechanism underlying TAZ-induced tumorigenesis, we tested the functional domains of TAZ important for TAZ-S89A-induced CSC and tumorigenic phenotypes. Although equal levels of TAZ-S89A wild-type and mutants were expressed in the presence of Dox (Figure 2A), mutation of the TEAD binding domain (TAZ-S89A-F52/53A) abolished TAZ-induced increased cell proliferation (Figure 2B and 2D), transformation (Figure 2E and 2F) and spheroid formation (Figure 2G and 2H), whereas mutation of the TAZ-S89A WW domain (TAZ-S89A-WWm) had no effect on these phenotypes (Figure 2C and 2E–2H). It seems that WW domain mutant only caused reduced sphere size (Figure 2I). These studies suggest that the TEAD binding domain rather than the WW domain of TAZ is essential for TAZ-S89A induced lung tumorigenesis and cancer stem cell phenotypes.

### Identification of cellular genes mediating TAZ-induced tumorigenesis and CSC phenotype

**Figure 3:**
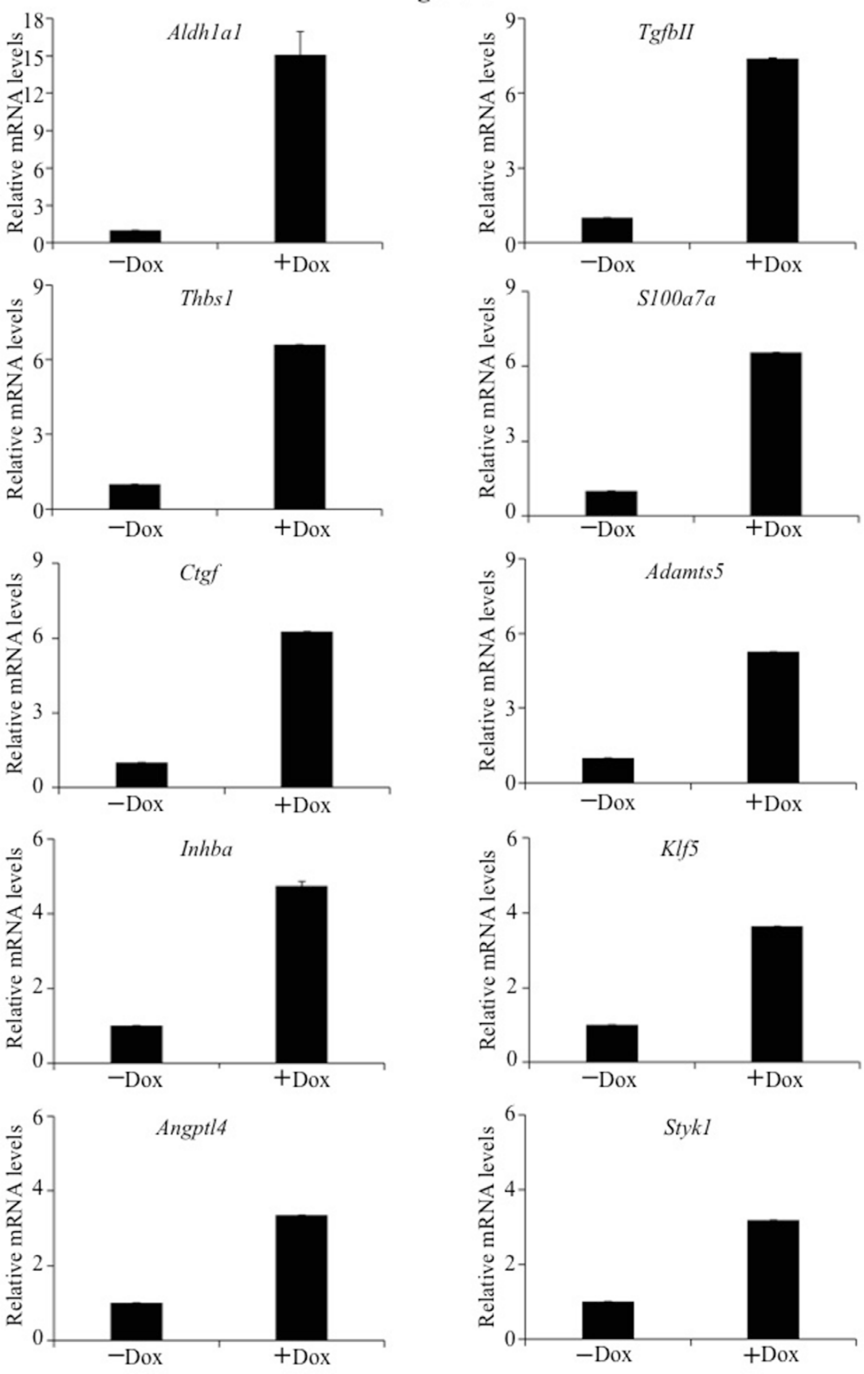
qRT-PCR confirmation of cellular genes activated by TAZ Real time qRT-PCR was performed to examine selected cellular gene expression in E10-TAZ-S89A-T cells in the absence (−) or presence (+) of Dox for 24 h. The mRNA levels of E10-TAZ-S89A-T+Dox relative to those of E10-TAZ-S89A-Dox cells are presented here. The mean and S.D. were calculated from Ct values of triplicate real time RT-PCRs for each RNA sample.

Since TAZ is a transcriptional coactivator, it may cause lung tumorigenesis and CSC phenotypes by transcriptionally activating downstream gene(s). To identify novel downstream genes transcriptionally upregulated by TAZ, gene expression profiles of E10-TAZ-S89A-T cells in the absence and presence of Dox induction were compared by RNA-sequencing (RNA-seq). After inducing TAZ-S89A for 24 hours, a total of 168 genes were found upregulated (Supplementary Table 1). qRT-PCR (quantitative reverse transcription PCR) analysis validated a total of 10 oncogenes that are upregulated at least 3 fold (Figure 3; Supplementary Table 4). Of these genes, human homologs of 4 genes including *Inhibin, beta A* (*INHBA*), *Kruppel-like factor 5* (*KLF5*), *Serine/threonine/tyrosine kinase 1* (*STYK1*), and stem cell marker *ALDH1A1* are previously shown to be over-expressed in NSCLC and involved in lung cancer progression and tumorigenicity [16, 20–23]. In this study, we have further characterized *Aldh1a1*, the most significantly up-regulated gene (Figure 3), as a *bona fide* downstream target of TAZ during lung tumorigenesis.

### Characterization of Aldh1a1 as a novel transcriptional target of TAZ

**Figure 4:**
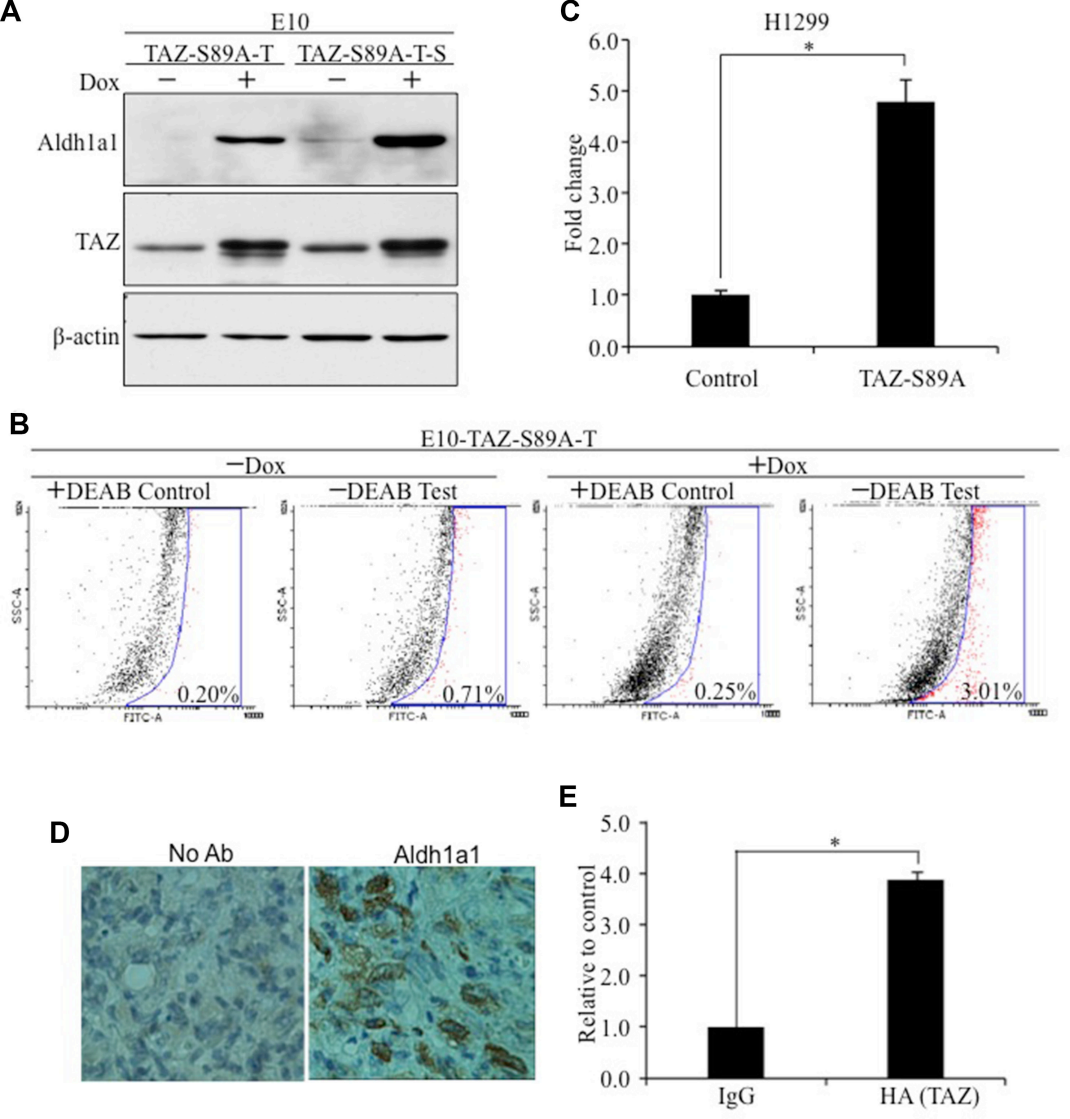
Characterization of Aldh1a1 as a novel transcriptional target of TAZ (**A**) Western blot analysis of ALDH1A1 expression. ALDH1A1 was detected by anti-ALDH1A1 (1:500, Abcam) antibody. (**B**) Flow cytometry analysis of ALDH1A1 levels by Aldefluor assay. E10-TAZ-S89A-T cells were cultured in the absence (−Dox) or presence (+Dox) of Dox for 2 days. Cells were untreated (−DEAB, Test) or treated (+DEAB, Control) with the ALDH1A1 enzyme inhibitor DEAB, followed by FACS analysis. The experiments were repeated 3 times. A representative set of data is shown here. (**C**) Overexpression of TAZ-S89A significantly increased *ALDH1A1* promoter activity. Aldh1a1-Gluc reporter (100 ng) was transiently transfected together with pcDNA 3.1 vector (Control, 400 ng) or TAZ-S89A-HA (400 ng) into H1299 lung cancer cells. CMV-β-galactosidase (100 ng) was also transfected as an internal control. After 24 hours, cells were harvested and tested for reporter activity by BioLux *Gaussia* Luciferase Assay Kit and β-galactosidase assay. The mean ±S.D. (*n* = 3) is shown. *t*-test, *P < 0.05. (**D**) Immunohistochemical analysis of Aldh1a1 expression in tumors formed after injection of E10-TAZS89A into nude mice. Anti-Aldh1a1 antibody (1: 200) dilution was used in the staining. No antibody was used as internal control. (**E**) ChIP analysis of the interaction of TAZ with ALDA1A1 promoter. qPCR was performed using primers flanking ALDH1As promoter and chromatins extracted from cells expressing TAZ-HA. The data is presented as the relative Ct value of ALDH1A1 qPCR curve from HA (TAZ)-precipitated to that of IgG (antibody control)-precipitated chromatin-DNA.

To further confirm Aldh1a1 is a novel downstream target of TAZ, we examined the protein levels of Aldh1a1 by WB after induction of TAZ-S89A by Dox. Aldh1a1 is significantly induced after TAZ-S89A overexpression (Figure 4A). Since TAZ-S89A-T cells form sphere like CSCs (Figure 1F), we also isolated the cells forming the lung spheres (TAZ-S89A-T-S). Significantly, we found that both TAZ and ALDH1A1 are higher in TAZ-S89A-T-S than TAZ-S89A-T cells (Figure 4A), suggesting that the level of ALDH1A1 is positively correlated with TAZ level and CSC phenotype. In addition, by using an Aldefluor assay to detect active Aldh1a1 in cells, we found that induction of TAZ-S89A by Dox caused over 4-fold (0.71% vs 3.01%) induction of active Aldh1a1 (Figure 4B). In addition, our further studies suggested that ALDH1A1 other than ALDH1A2 and ALDH1A3 is specifically activated by TAZS89A (Supplementary Figure 4). Therefore, although the ALDFLUOR assay generally detects all ALDH1A isoforms (ALDH1A1, 2, 3), enhanced ALDH1A1 is responsible for increased ALDH1 activity in cells after TAZ overexpression.

**Figure 5:**
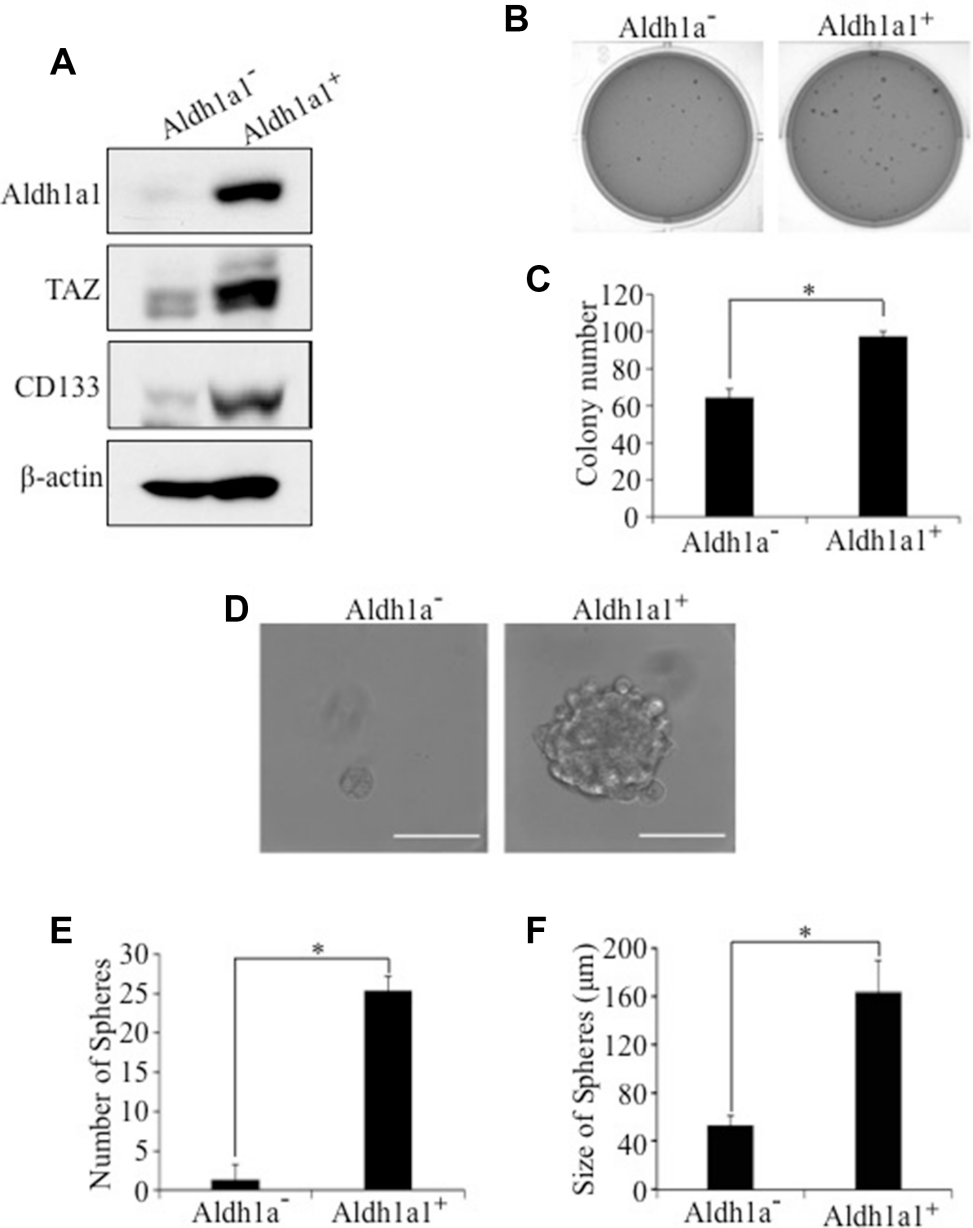
Enhanced tumorigenic and CSC phenotypes in Aldh1a1-positive E10-TAZS89A-T cells Aldh1a1-positve (Aldh1a^+^) and Aldh1a1-negative (Aldh1a^−^) cells were sorted from E10-TAZS89A-T cells by FACS. (**A**) Enhanced expression of TAZ and CD133 in Aldh1a1+ E10-TAZS89A-T cells. (**B**–**F**) Enhanced transformation (B, C), sphere number (E), and sphere size (D, F) in Aldh1a1^+^ E10-TAZS89A-T cells. The experimental procedures and statistic analysis were performed as described in Figure 1.

To test whether activation of *Aldh1a1* transcription by TAZ is due to activation of Aldh1a1 promoter, we performed a luciferase assay in H1299 human lung cancer cells using an *ALDH1A1* promoter *Gaussia* luciferase reporter (ALDH1A1-Gluc). Overexpression of TAZ-S89A significantly increased *ALDH1A1* promoter activity (Figure 4C). Most significantly, IHC analysis showed that Aldh1a1 is highly expressed tumor formed in mice injected with E10-TAZS89A-T cells (Figure 4D). In addition, TAZ directly interacts with ALDH1A1 promoter (Figure 4E). Enhanced levels of ALDH1A1 and tumorigenic and CSC phenotypes were also observed after overexpression of TAZS89A in HBE135 lung human epithelial cells (Supplementary Figure 2). Since Alpha1a1 positive (Aldh1a1^+^) CSC cells only accounts for around 3% of cell pollution, we isolated Aldh1a1^+^ cells from E10-TAZS89A-T cells to see whether transformation and CSC phenotype is indeed mostly caused by Aldh1a1+ cells. As expected, we data showed that the expression of TAZ and another lung CSC marker CD133 is significantly higher in Aldh1a1^+^ than Aldh1a1^−^ cells (Figure 5A). In addition, Aldh1a1+ cells have higher ability for transformation and sphere formation than Aldh1a1- cells (Figure 5B–5F).

**Figure 6:**
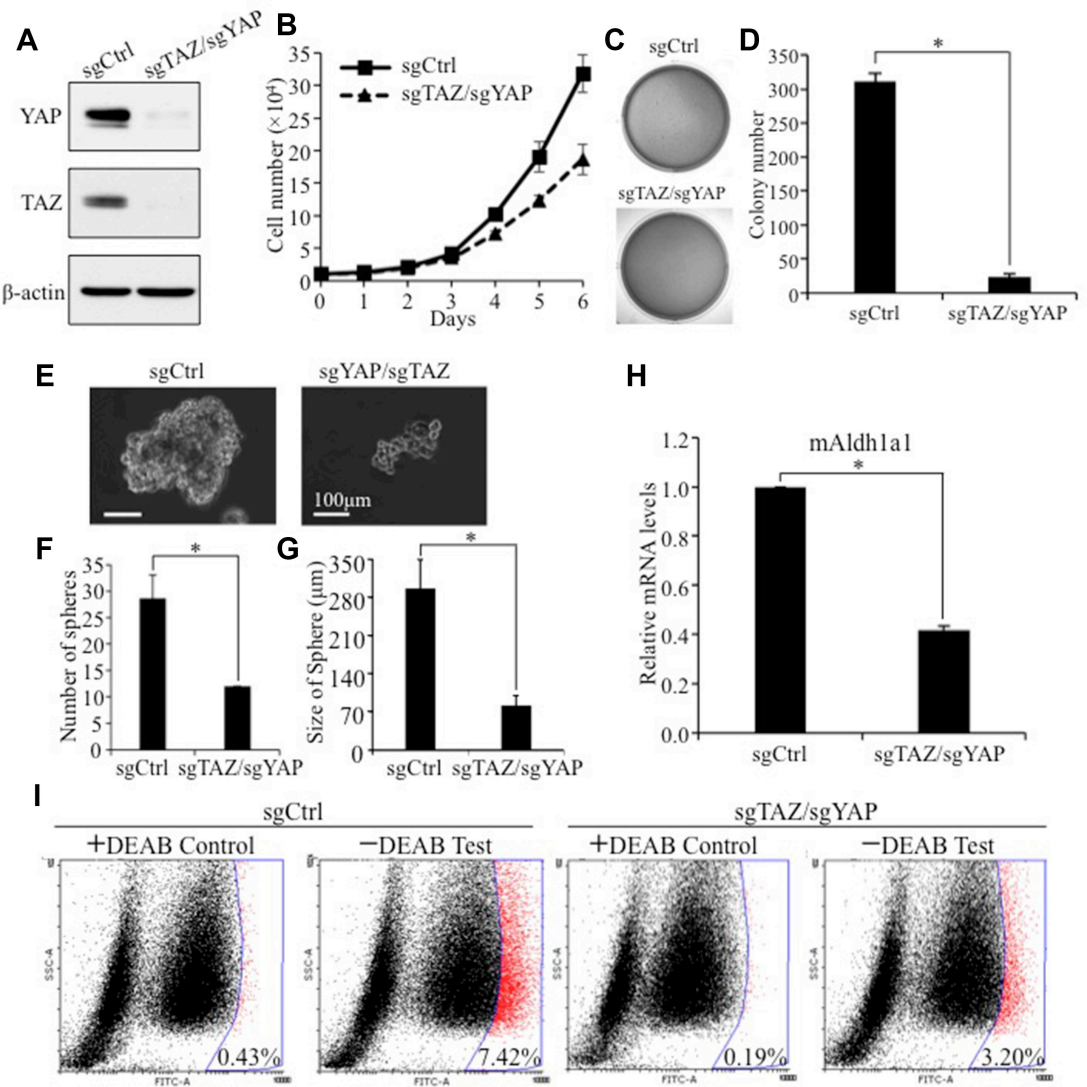
TAZ/YAP knockout inhibits human NSCLC cell anchorage-independent growth, CSC sphere formation, and ALDH1A1 activity levels **(A)** Western blot analysis of TAZ and YAP levels in A549 cells. By using two CRISPR sgRNAs targeting TAZ and YAP, both protein levels of TAZ and YAP were abolished in A549 cells. YAP and TAZ was detected by anti-YAP (1:1000, Santa Cruz) and anti-TAZ (1:1000; BD) antibodies, respectively. **(B)** TAZ/YAP knockout inhibits cell proliferation. Triplicate of 1 × 10^4^ A549 with TAZ/YAP knockout cells were seeded into each well of 12-well plates. Data are shown as means ±S.D., *n* = 3. **(C–D)** Soft-agar assay. Procedures and data analysis were as described in Figure 1D–1E. **(E–G)** Sphere formation assay. Experimental procedures and data analysis were as described in Figure 1F–1G. **(H)** qRT-PCR analysis of *ALDH1A1* mRNA after TAZ/YAP knockout. mRNA levels of *ALDH1A* in A549 cells expressing sgRNA vector (sgCtrl) or sgTAZ/sgYAP were analysis by qRT-PCR. The mRNA levels of A549-sgTAZ/sgYAP relative to those of A549-sgCtrl cells are presented here. **(I)** Aldefluor analysis of ALDH1A1 after TAZ/YAP double knockout in A549 cells. Procedures and data analysis were as described in Figure 4B.

Next, we wished to further confirm our result in other lung cell lines and test whether endogenous TAZ is essential for activation of Aldh1a1 in lung cancers. Since TAZ and YAP have redundant function, we simultaneously knocked out TAZ alone or in combination with its paralog YAP in TAZ/YAP-high and ALDH1A1-high A549 human lung cancer cells using two single-guide RNAs (sgRNAs) targeting TAZ and YAP (Figure 6A). Interestingly, TAZ single knockout have no or minor effect on Aldh1a1 levels, cell proliferation, transformation and CSC phenotypes (Supplementary Figure 3) probably due to suppressing and compensate effects of TAZ on YAP (Supplementary Figure 1). However, TAZ/YAP double knockout (sgTAZ/sgYAP) in these cells not only significantly reduced their proliferation (Figure 6B), transformation (Figure 6C and 6D) and CSC sphere formation (Figure 6E and 6F), but also caused significant reduction of *ALDH1A1* mRNA (Figure 6G) and protein (Figure 6H) in A549 cells. In addition, reduction of *ALDH1A1* mRNA was also found when TAZ and YAP are knocked out in another lung cancer cell line, H358 (data not shown). Together, our studies provide convincing evidence that *ALDH1A* is a major *bona fide* downstream target of TAZ/YAP in lung CSC phenotypes and tumorigenesis.

### TEAD-dependent activation of Aldh1a1 transcription by TAZ

**Figure 7:**
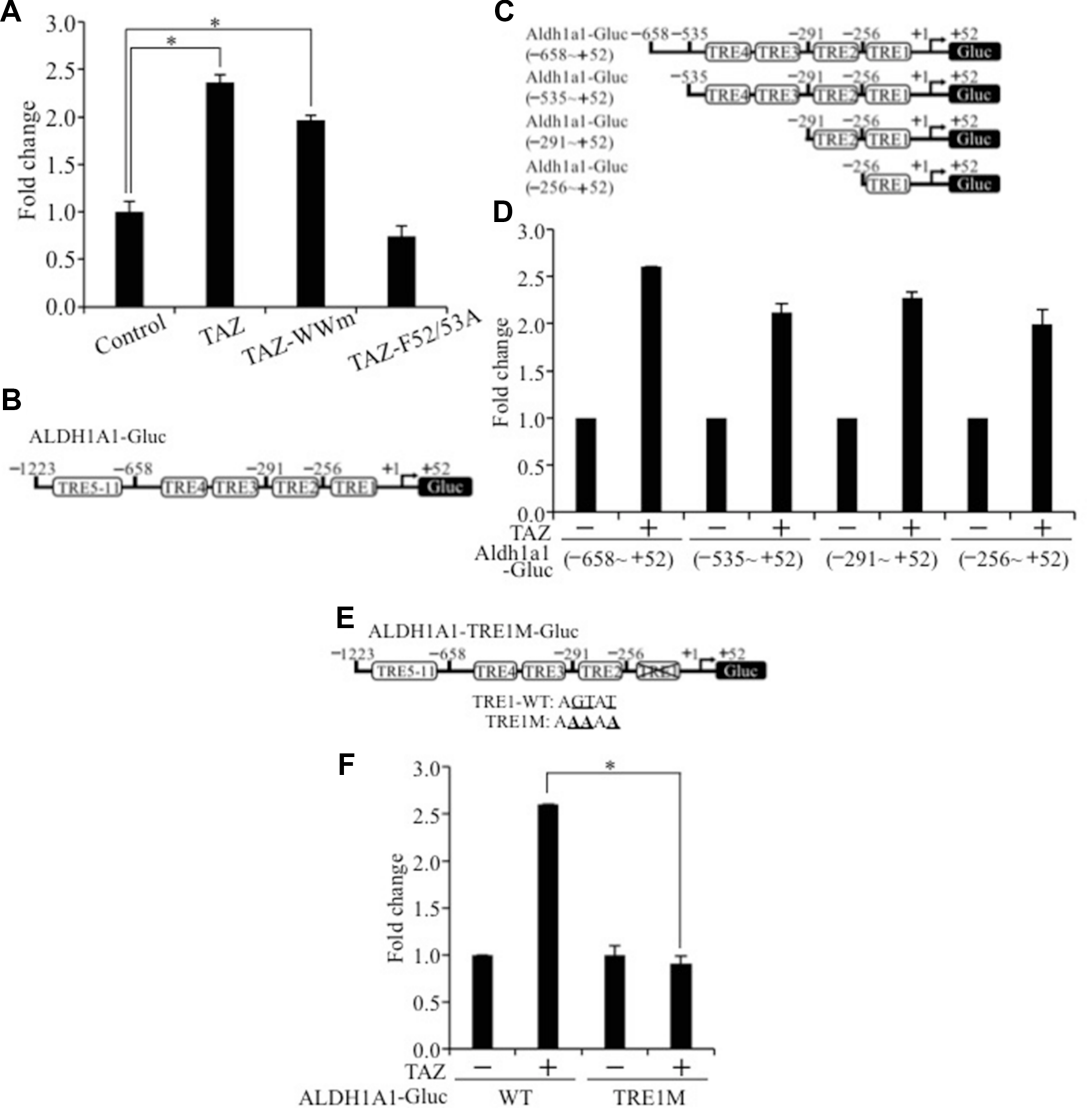
TEAD-dependent activation of the ALDH1A1 promoter by TAZ (**A**) Functional domain important for activation of *ALDH1A1* promoter. Aldh1a1-Gluc and b-galactosidase alone to together with wild-type TAZ, TAZ-WWm, or TAZ-F52/53A was transiently transfected into H1299 cells. Relative luciferase activity was determined as described in Figure 4C. (**B**) Eleven potential TEAD response elements (TREs) in the *ALDH1A1* promoter. The full length *ALDH1A1* promoter contained within the region of -1223~+52 and was cloned into GLuc vector. (**C**) Deletions of *ALDH1A1* promoter. (**D**) Luciferase analysis of *ALDH1A1* promoter deletions. (**E**) TRE1 mutation construct and sequences. Mutated nucleotides in TRE1 are bolded and underlined. (**F**) Luciferase analysis of the *ALDH1A1* promoter with TRE1 mutation. Procedure and data analysis were as described in Figure 4C.

Our findings suggest that interaction of TAZ with the TEAD transcription factor is essential for TAZ-S89A-induced lung tumorigenesis and CSC phenotypes (Figure 2A–2H). Therefore, we further tested whether TAZ activates *ALDH1A1* transcription through activation of TEAD. Our luciferase assay showed that wild-type TAZ or TAZ WW mutant (TAZ-WWm) significantly increased, whereas mutation of TEAD binding domain in TAZ (TAZ-F52/53A) completely abolished *ALDH1A1* promoter activity (Figure 7A). By examining the *ALDH1A1* promoter sequence, we found 11 putative TEAD response elements (TREs; Figure 7B). Deletion analysis showed that TRE1 localized in the -256 ~ +52 region of *ALDH1A1* promoter is responsible for the majority of its activation by TAZ (Figure 7C–7D). Since only a single TRE1 is located in -256 ~ +52 region of *ALDH1A1* promoter, we further mutated the TRE1 (TRE1M) in the full-length *ALDH1A1* promoter and showed that TRE1 mutation completely abolished its activition by TAZ (Figure 7E and 7F). In summary, these results strongly suggest that TAZ co-activator up-regulates *ALDH1A1* by directly interacting with and activating the *ALDH1A1* promoter through the TEAD transcriptional factor.

### Activation of Aldh1a1 is important for TAZ-induced tumorigenesis and CSC phenotype

**Figure 8:**
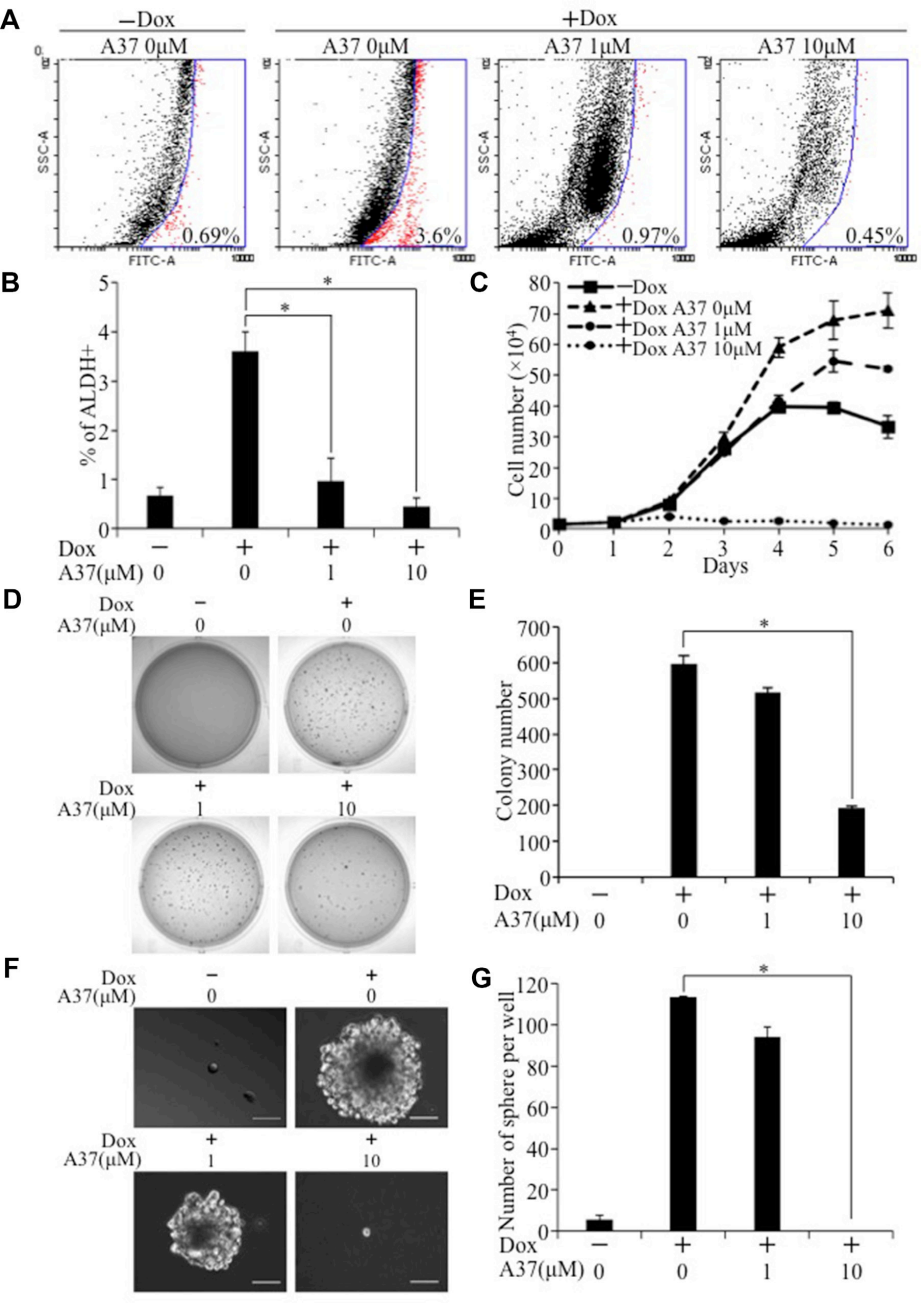
Inhibition of TAZ-S89A-induced tumorigenic and CSC phenotypes by an Aldh1a1 inhibitor E10-TAZ-S89A-T cells were cultured in the absence (Dox-) or presence of Dox (Dox+) with increasing concentration of A37 (0, 1, 10 μM) for 2 days for Alfdefluor assay (**A**, **B**), 6 days for cell proliferation assay (**C**), 18 days for soft agar assay (**D**, **E**), or 7 days for sphere formation assay (**F**, **G**). Experimental procedures and data analysis were as described in Figure 1A–1G and Figure 4B.

**Figure 9:**
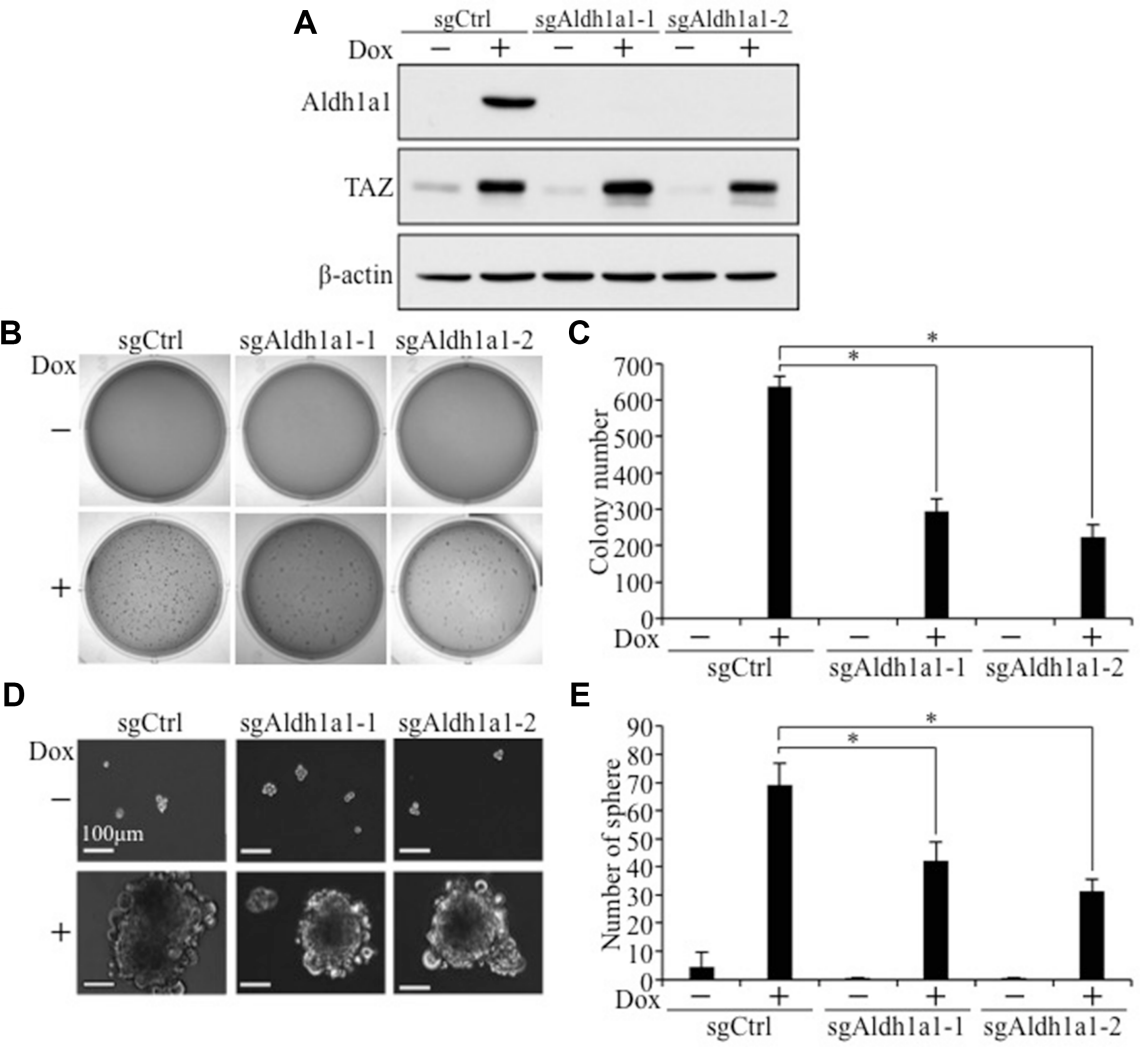
*Aldh1a1* knockout significantly inhibits TAZ-S89A-induced tumorigenic and CSC phenotypes (**A**) Western blot analysis of ALDH1A1 after *Aldh1a1* knockout by CRISPR. E10-TAZ-S89A-T expressing sgCtrl or two different sgAldh1a1 (sgAldh1a1-1 and sgAldh1a1-2) were cultured in the absence (−) or presence (+) Dox for 2 days, followed by protein extraction and WB analysis. (**B**–**E**) Aldh1a1 knockout reduced TAZ-induced anchorage-independent growth (B, C) and sphere formation (D, E). E10-TAZ-S89A-T cells expressing sgCtrl or sgAldh1a1-1 or sgAldh1a1-2 were untreated (−) or treated with Dox (+) for 18 days (soft-agar) or 7 days (sphere formation assay). All procedures and data analysis were as described in Figure 1.

To examine whether activation of Aldh1a1 is critical for TAZ-induced tumorigenesis and cancer stem cell phenotypes, we first treated E10-TAZ-S89A-T cells with increasing concentrations of A37, an Aldh1a1-specific inhibitor recently developed and used by others [24]. While treatment of E10-TAZ-S89A-T cells with 1 μM of A37 reduced active ALDH1A1 (Figure 8A, 8B), which also lead to reduced cell proliferation (Figure 8C), transformation (Figure 8D, 8E), and CSC sphere size (Figure 8F) and number (Figure 8G), treatment of these cells with 10 μM of A37 completely abolished the above phenotypes (Figure 8A–8G). To exlude the possibility that a high concentration of this small molecule drug may have non-specific effect, we also completely knocked out Aldh1a1 in E10-TAZ-S89A-T cells using two sgRNAs against different regions of the *Aldh1a1* gene (sgAdlh1a1-1 and sgAdlh1a1-2; Figure 9). Knockout of *Aldh1a1* significantly reduced TAZ-induced transformation (Figure 9B, 9C) and CSC phenotype (sphere formation) (Figure 9D and 9E). To confirm these findings *in vivo*, we injected low cell numbers (1 × 10^4^ cells) of E10-TAZ-S89A-T and E10-TAZ-S89A-T-sgAdlh1a1 cells into nude mice, followed by treating their drinking water with (+Dox) or without (−Dox) Dox. Tumor formation *in vivo* was observed in the majority of mice (4 out 5) when E10-TAZ-S89A-T cells were injected, whereas knockout of *Aldh1a1* in E10-TAZ-S89A-T cells (E10-TAZ-S89A-sgAdhl1a1) completely abolished tumor seeding and growth in mice (Table 1). Together, these *in vitro* and *in vivo* studies strongly suggest that *Aldh1a1* is a critical gene mediating TAZ-induced lung tumorigenesis and CSC phenotyptes.

**Table 1:** Summary of tumorigenicity analysis

Cell lines	Injected cell number	Dox	Tumor formation
E10-TAZS89A-T-sgCtrl	1 × 10^4^	−	0/5
		+	4/5
E10-TAZS89A-T-sgAldh1a1-1	1 × 10^4^	−	0/5
		+	0/5
E10-TAZS89A-T-sgAldh1a1-2	1 × 10^4^	−	0/5
		+	0/5

## DISCUSSION

Mounting evidence suggests that CSCs are critical for lung tumorigenesis, metastasis, and resistance to therapeutic drugs. Therefore, targeting the CSC for lung cancer therapy has become one of the hottest areas in cancer research and therapy [25–27]. However, although many genes have been identified that are involved in lung tumorigenesis, there are few cancer-causing genes shown to be essential for lung CSC phenotypes. Our TAZ overexpression and knockout studies *in vitro* and *in vivo* strongly suggest that TAZ is not only a driver for lung CSC formation but also essential for the maintenance of the CSC phenotype in lung cancers (Figures 1 and 6). Therefore, TAZ can be a novel target to specifically inhibit CSC growth for more effectively treating early-stage, metastatic, and drug resistant lung cancer in the future. In addition, since only constitutively active TAZ, which lacks the inhibition by the upstream inhibitor LATS1/2, can induce cancer stem cell and tumorigenic phenotype in lung epithelial cells, dysregulation of other components of the Hippo pathway may also play important role in lung tumorigenesis [4], TAZ-TEAD-Aldh1a1 may be a critical signaling axis in mediating CSC formation and tumorigenesis caused by dysregulation of other components of the pathway, and therefore a potential target for cancers with dysregulation of the Hippo pathway.

Although Aldh1a1 has well been regarded as a standard CSC marker (14), why it is upregulated in CSC is still unclear. This study showed that TAZ is a critical upstream activator of Aldh1a1 in lung CSCs. Since TAZ is also involved in the formation of CSC in other cancers such as breast cancer [11, 12, 28], our findings provide the first molecular mechanism underlying enhanced levels of Aldh1a1 in CSCs. In addition, our studies also indicate that Aldh1a1 is not only a marker, it also mediates TAZ-induced lung CSC phenotypes and tumorigenesis. We showed that inactivation of Aldh1a1 by its inhibitor or CRISPR (Clustered Regularly-Interspaced Panlindromic Repeat) gene editing can block TAZ-induced lung tumorigenic and CSC phenotypes *in vitro* and tumor formation *in vivo* (Figures 8 and 9) . However, knockout of other TAZ downstream lung oncogenes such as *Inhba*, *Klf5*, or *Styk1* (Figure 3) by CRISPR has no effect (data not shown). This study strongly suggests that ALDH1A1 is a critical mediator of TAZ-induced CSC phenotypes and tumorigenesis. Most significantly, since overexpression of TAZ is found in over 60% lung cancer [8, 9], inhibiting TAZ or ALDH1A1 alone or in combination may provide a more effective therapy for the treatment of TAZ-associated lung cancer in the future.

Conflicting data regarding the roles of ALDH1A1 in lung CSC and tumorigenesis has been reported previously. In one study, ALDH1A1 expression was found to be associated with favourable prognosis [29], whereas in other studies enhance levels of ALDH1A1 was associated with severe tumor phenotypes and poor prognosis [30, 31]. However, our functional studies strongly suggest that ALDH1A1 is an oncogene and CSC marker in mediating tumorigenic and CSC function of TAZ oncogene (Figures 7 and 8) and ALDH1A1-positive lung epithelial cells obtained higher tumorigenic and CSC potential than ALDH1A1-negative cells (Figure 5). Consistent with our findings, it has also been shown by others that overexpression of ALDH1A1 rather than other isoforms (ALDH1A3 and ALDH3A1) increased lung cancer cell transformation and CSC phenotype by activating stem cell drivers Nanog, Oct4, and Sox2 [32]. On the other hand, knockdown of Aldh1a1 in CSCs results in reduced activity of oncoproteins Akt and GSK3beta [33]. Therefore, it is possible that Aldh1a1 mediates TAZ oncogenic function in CSC phenotypes and lung tumor development by upregulating these stem cell markers or/and Akt/GSK3beta. However, we also noticed that although TAZ-S89A is overexpressed in all E10-TAZ-S89A-T cells, only low percentage (3.01%) of them are positive for ALADH1A (Figure 4B), suggestint that TAZ-S89A may specifically activate *ALDH1A1* in CSCs. We have confirm this by showing that Aldh1a1+ cells isolated from mix polulation of E10-TAZS89A-T cells have stronger transformation and CSC ability (Figure 5A–5F). It will be very interesting to further investigate how ALDH1A1 is only activated by TAZ in CSCs and whether high levels of TAZ is positively correlated with those of ALDH1A1 (CSC-specific) in clinical lung cancer patients.

In conclusion, this study provides convincing evidence that TAZ is a driver for lung tumorigenesis *in vivo* and a novel regulator of lung CSCs and that TAZ regulates these processes through upregulation of Aldh1a1. These findings will have significant implications for the prognosis, diagnosis, and therapy of lung cancer in the future.

## MATERIALS AND METHODS

### Plasmid construction and site-directed mutagenesis

Full-length cDNA of constitutively active human TAZ-S89A was first amplified by PCR using the following primers: pTRIPZ-TAZ-S89A-F: 5′-ATACCGGTACCATGAATCCG GCCTCG G CG-3′ and pTRIPZ-TAZ-S89A-R: 5′-CATACGCGTTTA TGCGTAGTCTGGGACATCGTAT GGATACAGCCAG GTTAGAAAG-3′. The PCR product was digested with AgeI/MluI and subcloned into the Dox-inducible lentiviral vector pTRIPZ (Open Biosystems). The human *ALDH1A1* promoter Gaussia luciferase reporter (Aldh1a1-Gluc) was purchased from GeneCopoeia (USA). Deletions of the promoter were constructed by PCR and site-directed mutagenesis using primers as the following: Aldh1a1-(−658~+52)-Gluc-F: 5′-CCGAATTCCCTAAAAGTCCTG-3′; Aldh1a1-(−535~+52)-Gluc-F:5′-CCGAATTCGTCTGT CAGAGAAC AGAAAG-3′; Aldh1a1-(−291~+52)-Gluc-F: 5′-CCGA ATTCTAACTGGCCTTA GTGGCCAG-3′; Aldh1a1- (−256~+52)-Gluc-F: 5′-CCGAATTCCACTTATCACAGG TTTCGG C-3′, and Aldh1a1-Gluc-R:5′-CACAAGCT TTTC TGATTCGGCTCCTGGAAC-3′. Mutations were constructed with following primers: Aldh1a1-TRE1M-Gluc-F:5′-CTGAGTTTGTTCAT CCAATCGTATCCGAA AAAGCAAATAAACTTTAGCCCGT-3′ and Aldh1a1- TRE1M-Gluc-R: 5′-ACGGGCTAAAGTTTATTTG CTT TTTCGGATACGATTGGATGAACAAACTCAG-3′. PCR products were digested by EcoRI/HindIII, followed by subcloning back into the pEZX-PG02-Gluc reporter vector as described [34]. TRE1 sequence was mutated from AGTAT to AAAAA by either the Quick-Change Mutagenesis kit (Strategene) or overlapping PCR mutagenesis.

### Cell culture

E10 (mouse immortalized lung epithelial cell line), A549 (adenocarcinomic human lung epithelial cell line), and HEK293T (human embryonic kidney cell line) were cultured in Dulbecco's Modified Eagle's Medium (DMEM; Sigma, #D6429) containing 10% fetal bovine serum (FBS), and 1% penicillin/ streptomycin (P/S) (Invitrogen). H358 (human NSCLC cell line) and H1299 (human NSCLC cell line) were maintained in RPMI-1640 medium (Sigma, #8758) containing 10% FBS and 1% P/S supplemented with Sodium Pyruvate (1mM), HEPES (10mM) and Glucose (2.5mg/ml). All cells were maintained at 37°C with 5% CO_2_.

### Lentivirus production, concentration, and infection

Twenty-four hours before transfection, 2´10^6^ 293T (passage lower than 10) cells were seeded in 60mm tissue culture dishes pre-coated with 0.1 mg/ml poly-L-lysine and incubated at 37°C overnight. Then, 1 μg of lentiviral constructs were mixed with 0.75 μg of psPAX (packaging), 0.25 μg of pMD2G (envelop) plasmids and 6 μl of PolyJet reagent (SignaGen) in serum-free medium. After incubation for 15 minutes at room temperature, the mixture was added dropwise into each plate. Twenty to twenty-four hours after transfection, the medium was replaced with 2 ml of DMEM/10%FBS containing 10 mM Na butyrate (demethylation of plasmids to increase gene expression). Two days after transfection, the media containing lentivirus were collected for direct viral infection or viral concentration using Lenti-X concentrator (Clontech) according to the manufacturer's protocol.

For lentivirus infection, 4–5 × 10^4^ cells were seeded into each well of 6-well plates. One day after plating, 8 μg/ml of polybrene and a series amount of virus were added into each well. Two days after infection and incubation at 37°C with 5% CO_2_, the cells were treated with 2 μg/ml puromycin for selection. After stable cell lines were established, cells were either collected for protein analysis, functional assays or tumorigenesis assay (see below).

### Gene knockout by CRISPR

For TAZ/YAP or Aldh1a1 gene knockout, 1–2 guided RNAs (gRNAs) sequences targeting TAZ/YAP or Aldh1a1 were chosen (Supplementary Table 2) based on published whole-genome gRNA libraray sequences [35, 36] and single-stranded complementary oligos with BsmBI overhang were synthesized (McGill University). FastDigest BsmBI and FastAP from Fermentas were used to digest LentiCRISPR v1 (Addgene) lentiviral vector. Digested vectors were purified by QIAquick Gel Extraction Kit and eluted in EB buffer. Oligos were phosphorylated and annealed by T4 polynucleotide kinase (PNK, NEB, #M0201S) in T4 ligation Buffer (NEB) following a thermocycler running at 37°C for 30 minutes, 90°C for 5 minutes and then ramp down to 25°C at 5°C per minute. Ligation was catalyzed by mixing annealed oligos and digested LentiCRIPSR v1 vector with Quick Ligase in Quick Ligase Buffer (NEB, #M2200S), followed by transformation into Stbl3 bacteria. Production of lentivirus and establishment of stable cell lines were as described above.

### Protein extraction, antibodies, and western blot

Mammalian cell protein extraction and WB were as described [34]. Protein lysates were equally adjusted to 10–20 μg for TAZ, YAP, Aldh1a1 and β-actin detection. Primary antibodies were used as the following: mouse monoclonal anti-TAZ (1:1000) antibody from BD Biosciences; rabbit polyclonal antibody YAP (H125, 1:1000) from Santa Cruz and CD133 from protein tech (1:500); rabbit monoclonal anti-Aldh1a1 (1:500) antibody from Abcam and mouse monoclonal anti-β-actin (1:10,000) from Sigma.

### RNA extraction, RNA-seq, qRT-PCR

For total RNA extraction, cells were cultured until 70–80% confluency following by total RNA extraction through RNAzol^®^RT reagent (Molecular Research Center, Inc., Cincinnati, OH, USA) according to the manufacturer's protocol. For RNA-seq purposes, RNA extracted from E10-TAZ-S89A-T cells treated with or without Dox was further purified using the RNeasy Mini Kit (Qiagen), prepared as equal aliquots (3μg), and subsequently sent to BGI Americas Corporation for RNA-seq quantitation and analysis. Based on their differential expressions (≥ 2-fold increase) and oncogenic significances, genes of interests were selected and further confirmed by qRT-PCR.

For qRT-PCR, SuperScript III Platinum SYBR Green One-Step qRT-PCR Kit (Invitrogen) was used for real-time qRT-PCR to validate the level of each differentially expressed gene. Real time qRT-PCR analysis was performed as described [34]. In brief, a duplicate of 50ng of total RNA extracted from cells were mixed with specific primers Supplementary Table 3, followed by running on the ViiA 7 Real-Time PCR System. RT-PCR was run at 1 cycle of 50°C for 10 minutes, 95°C for 5 minutes and 40 cycles of 95°C for 10 seconds, 60°C for 30 seconds. 18S rRNA was used as internal control of RNA. The relative mRNA level of each gene was calculated as the following formula: 2^−(gene^ ∆^CT – Control^ ∆^CT)^, where ∆CT = gene average C_T_ - rRNA average C_T_. Gene expression levels were compared between cells infected with lentivirus expressing vector control (A549 and H358) or without Dox-induced TAZ overexpression (E10-TAZ-S89A-T-Dox) and those infected with lentivirus expressing sgTAZ/sgYAP or sgAdhla1 or Dox (+). The mean and S.D. were calculated from Ct values of duplicate real-time RT-PCR. Student *t*-test was used for statistical analysis of the difference between control (Dox- or sgCtrl) and test (Dox+ or sgTAZ/sgYAP or sgAldh1a1) group. *P* < 0.05 was considered as statistically significant. The same statistical analysis was used for soft-agar, sphere formation, Aldefluor, and luciferase assays described below.

### *Gaussia* luciferase and β-galactosidase assays

Triplicates of 1 × 10^5^ H1299 cells were plated in 12-well plates 24 hours before transfection. After changing fresh complete growth media, cells were transiently transfected with Aldh1a1-Gluc reporter or its mutants (100 ng) alone or together with vector control pcDNA3.1, TAZ-S89A-HA, TAZ-HA, TAZ-WWm-HA, TAZ-F52/53A-HA (400ng) using PolyJet at a ratio of 3:1 (volume of PolyJet to mass of plasmid). As an internal transfection control, CMV-β-galactosidase (100 ng) was also co-transfected in each sample. After 24 hours, cells were harvested and measured for *Gaussia* Luciferase activity by BioLux Gaussia Luciferase Assay Kit using the Gaussia Luciferase Reporter Assay System (Promega) and Turner Biosystems 20/20 luminometer. β-galactosidase assay was started by mixing 30μl cell lysates with MgCl_2_ (0.1M, in 4.5M beta-mercaptoethanol), *o*-nitrophenyl-β-D-galactoside (ONPG, 4mg/ml, pH=7.5), and sodium phosphate (0.1M, pH=7.5). After incubation at 37°C for about 10 min or until a faint yellow color developed, the reactions were stopped by adding 500 μl Na_2_CO_3_ (1M). Mixtures were transferred to each well of a 96-well plate and color intensities were measured at a wavelength of 420 nm by VersaMax Microplate Reader (Molecular Devices, LLC., USA). β-galactosidase activity (units/ml) = A420 × (0.0045)^−1^ × (reaction time (min) × cell extraction volume (ul))^−1^. Luciferase activity of each sample was calibrated with β-galactosidase activity. The fold change was calculated based on the ratio of averaged luciferase activity of ALADH1A-Gluc or its mutants after transfection of TAZ/TAZ-S89A or its mutants to that after transfection of vector (control). The experiments were repeated 3 times. The means ± SD of each transfection were shown.

### ChIP analysis

ChIP analysis was performed to detect interaction of TAZ and ALDH1A1 promoter in cells. In brief, ALDH1A1 promoter construct was co-transfected with TAZ-S89A-HA plasmid into HEK293 cells, followed by treatment with 1% formaldehyde for 10 min, lysed in ChIP buffer (50 mM Tris pH 7.5, 150 mM NaCl, 5 mM EDTA, 0.5% NP-40, 1% Triton-X-100, 0.1% SDS). DNA was sheared by sonication and the sheared chromatin was incubated with 1:50 dilution of CHIP-specific anti-HA rabbit monoclonal antibody (Cell Signaling) or control mouse IgG, followed by qPCR using the primers used for amplification of the ALDH1A1 promoter. The binding of TAZ-HA to ALDH1A1 promoter is estimated by calculating the fold increase of ALDH1A1 promoter DNA amplified from HA-precipitated chromatin in comparison of that from IgG (control) precipitated chromatin.

### Cell proliferation assay

Cell proliferation assay was as described [8, 37]. A triplicate of about 1.5 × 10^4^ E10 cells or 1 × 10^4^ A549 were seeded into each well of 12-well plates. Dox (doxycycline hyclate, 1 μg/ml, BioShop, Canada) was added into the cells on the next day. Cell numbers were counted on days 1, 2, 3, 4, 5, and 6 after plating. Dox was refreshed every two days by changing fresh culture media with Dox. Data are shown as means ± S.D. and experiments are repeated at least three times.

### Soft-agar assay

Soft agar assay was as described [34] A triplicate of about 2 × 10^3^ E10 or 2 × 10^4^ A549 cells were mixed with complete growth media containing 0.4% agarose and then overlaid on 0.8% agarose in each well of 6-well plates. On the next day, 1ml of complete growth medium with/without 2 μg/ml Dox was added on top of the agarose. Medium was refreshed every two or three days. After culturing for 18 days, colonies were stained with 0.005% crystal violet in 20% methanol. Pictures were obtained under white light by the Bio-Rad Gel Doc System (Bio-Rad, Mississauga, Canada) and colonies were quantified by colony count program in Quantity One software. Data are shown as means ± S.D. These experiments were repeated at least three times.

### Sphere formation assay

When E10 overexpressing TAZ or its mutants or A549 cells with or without TAZ/YAP knockout reached around 80% confluence, they were dissociated by trypsin-EDTA into single-cell suspensions. Triplicate of 1 × 10^4^ cells were suspended in Dulbecco's Modified Eagle Medium/F12 (Sigma-Aldrich Co. LLC., #D6421) supplied with L-glutamine, Epidermal Growth Factor (EGF, 20 ng/ml, Sigma-Aldrich Co. LLC., #E5036), Basic Fibroblast Growth Factor (bFGF, 20 ng/ml, Recombination human protein, AA10-155, Life Technology), and Insulin (4 μg/ml; Life Technologies, #A11429IJ). Cells were then seeded into each well of ultra-low-attachment 6-well plates (Corning, Inc., NY, USA). Following 7 days in culture, spheres that larger than 100 μm were quantitated and pictures were taken using TE200 Nikon Inverted Fluorescent Microscope (Nikon, Montreal, Canada). The diameter of each sphere is measured and average of diameter for each samples is regarded as sphere size.

### Aldefluor assay and Aldh1a1 cell sorting

To profile cells with high ALDH enzymatic activity, the Aldefluor kit (Stem Cell Technologies, Vancouver, BC, Canada) was utilized according to the manufacturer's instructions. Around 2 × 10^5^ cells were collected and suspended in Aldefluor Assay Buffer. Cells were incubated in ALDH substrate BODIPY-aminoacetaldehyde (BAAA). Cells that were able to take BAAA and catalyze BAAA to a fluorescent product BODIPY–aminoacetate (BAA), were considered as ALDH+ cells (−DEAB Test). Half of the cells was incubated under identical conditions with addition of the specific ALDH inhibitor diethylaminobenzaldehyde (DEAB) as a control (+DEAB control). Flow cytometry was conducted by MACSQuant Analyzer (Miltenyi Biotec Inc., San Diego, CA, USA). Data were analyzed by Flowing Software (http://www.us.konaskel.fi/flowingsoftware/).

To sort Aldh1a1 positive cells, about 1 × 10^6^ Dox-induced E10-TAZS89A-T cells were subjected to ALDH staining or unstrained (control) as described above. ALDH1A1^+^ cells were sorted using ALDH+DEAB as background ALDH1A1 negative (ALDH1A1^−^) control. The ALDH1A^+^ or ALDH1A^−^ cells were divided to extract protein for WB analysis or subjected to soft agar and sphere assays.

### Treatment of cells by ALDH1A1 inhibitor

E10-TAZ-S89A-T cells were treated with increasing concentrations (0, 1, and 10 μM) of ALDH1A1 inhibitor A37 for cell proliferation assay (6 days), sphere formation assay (7 days), soft-agar assay (18 days). For Aldefluor assay, cells were pre-treated with A37 for two days, followed by cell collection, reagents incubation and flow cytometry as described above.

### Tumorigenecity assay

For establishment of the TAZ-overexpressing tumorigenic cell line (E10-TAZ-S89A-T), about 3 × 10^6^ Dox-induced TAZ-overexpressing E10 cells were suspended in 100 μl 1 × PBS and subcutaneously injected into NU/NU nude mice (Jackson Lab). Mice injected with E10-TAZ-S89A-T were fed on normal condition as a control. Others were fed with food containing Dox to induce TAZ expression. The mice were watched for tumor formation for up to 10 weeks. The mice that showed tumor formation were dissected to isolate the tumor. The tumor was cut into small pieces, washed by 1 × PBS containing 1% P/S and trypsinized (1 ml trypsin/100 mg tumor) at 4°C overnight. On the next day, the trypsinized tissues were warmed at 37°C for 30 minutes, resuspended in culture media and passed through a 70 μm filter to get individual cells. Then cells were cultured in a 100mm plate with Ciprofloxacin to get rid of potential bacteria and Puromycin (1 μg/ml) to keep a selection pressure, sequentially.

For CSC tumorigenic assay, low cell numbers of 1 × 10^4^ tumorigenic E10-TAZ-S89A-T cells expressing sgCtrl or sgAldh1a1 were subcutaneously injected into each side of five mice, age matched between 5 to 6 weeks. Mice were fed with water without or with Dox (1 μg/ml) with refreshing on every other day. After 11 weeks, mice were euthanized.

### Immunohistochemistry analysis

Formalin fixed paraffin embedded (FFPE) tumor tissues were sectioned at the thickness of 3–4 μm, stained by the Discovery XT Automated IHC/ISH research slide staining system (Ventana Medical Systems, Inc.). Antigens were retrieved with EDTA (pH = 8) solution, blocked by 1% Bovine Serum Albumin (BSA, Fraction V), and incubated with mouse monoclonal anti-TAZ antibody (1:300 dilution, BD Biosciences) and rabbit monoclonal anti-ALDH1A1 (1:50 dilution, Abcam) antibody. As a control for specificity, one slide was processed with the same IHC condition except that primary antibody was not added. IHC signals were developed by using biotinylated HRP-conjugated anti-mouse or anti-rabbit secondary antibody, respectively, followed by catalyzing 3,3′-diaminobenzidine (DAB) substrate-chromogen into a visible precipitate. Pictures were taken using TE200 Nikon Inverted Fluorescent Microscope at 40× magnification.

## SUPPLEMENTARY MATERIALS FIGURES AND TABLES




