# Supplementary material for: TAZ induces lung cancer stem cell properties and tumorigenesis by up-regulating ALDH1A1
Source: Oncotarget. 2017 Mar 21;8(24):38426–43. doi: 10.18632/oncotarget.16430 (PMC5503543; doi:10.18632/oncotarget.16430)
Supplement: Supplementary file 1 [file oncotarget-08-38426-s001.pdf]

TAZ induces lung cancer stem cell properties and tumorigenesis by up-regulating ALDH1A1

Supplementary Materials

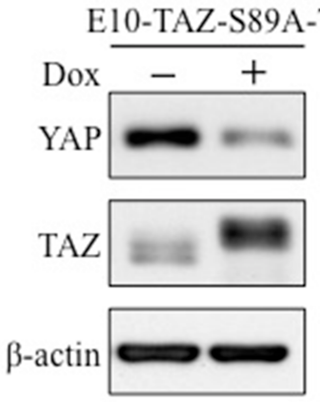

Supplementary Figure 1: Overexpression of TAZ in E10 cells suppresses YAP expression. Western blot analysis of TAZ and YAP expression. β-actin was used as an internal loading control.

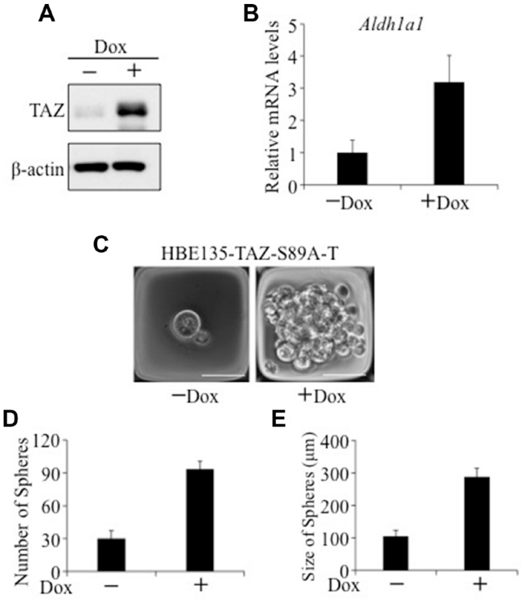

Supplementary Figure 2: Overexpression of TAZS89A in HBE135 cells increased Aldh1a1 expression and CSC phenotypes. Cells (HBE135-TAZS89A-T) derived from tumor formed after injection of HBE135-TAZS89A cells into nude mice were subjected to various essays in the absence (-) or presence (+) of Dox. (**A**) WB analysis of TAZ expression. (**B**) qRT-PCR analysis of ALDH1A1 mRNA levels. (**C**) Morphology of spheres of HBE135-TAZS89A-T cells. (**D**) Quantification of sphere numbers of HBE135-TAZS89A-T. (**E**). Quantification of sphere size.

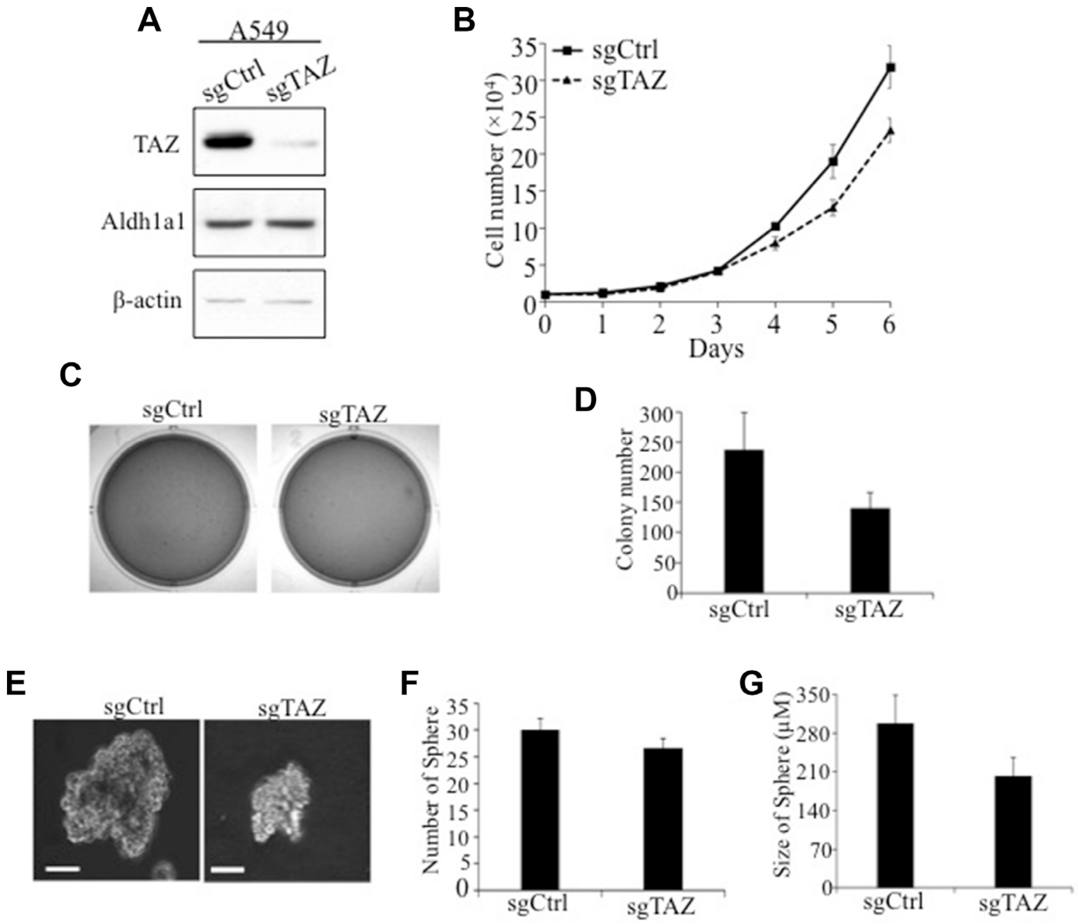

Supplementary Figure 3: Effect of TAZ knockout by sgRNA on tumorigenic and stem cell phenotypes. (**A**) Western blot analysis of TAZ expression. (**B**) Cell proliferation analysis. (**C**) Soft-agar assay. (**D**) Quantification of soft-agar assay. (**E**) Sphere formation assay. (**F**) Quantification of sphere number. (**G**) Quantification of sphere size.

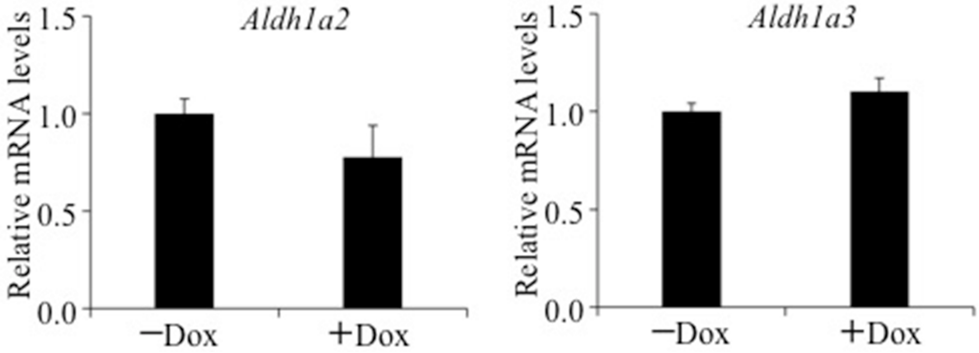

Supplementary Figure 4: Overexpression of TAZ ha no effect on mRNA expression of other isoforms of Aldh1a. qRT-PCR analysis of Aldh1a2 and Aldh1a3 mRNAs.

Supplementary Table 1: Cellular genes upregulated by TAZ-S89A overexpression.
See Supplementary_Table_1

Supplementary Table 2: Oligo sequences of gRNAs

sgRNA Oligos	Forward Primer (5′–3′)	Reverse Primer (5′–3′)
**sgTAZ**	CACCGTGCGCCAAGAGGAGCTCATG	AAACCATGAGCTCCTCTTGGCGCAC
**sgYAP**	CACCGTAATAGGCCAGTACTGATGC	AAACGCATCAGTACTGGCCTATTAC
**sgAldh1a1-1**	CACCGAAAGCATTAAAGTACTGTGC	AAACGCACAGTACTTTAATGCTTTC
**sgAldh1a1-2**	CACCGGAGAACTTACGGGGATGATT	AAACAATCATCCCCGTAAGTTCTCC

Supplementary Table 3: qRT-PCR primer sequences

Gene	Forward Primer (5′–3′)	Reverse Primer (5′–3′)
**hTAZ**	GAACATCAATCCCCAACAGACCCG	TTACAGCCAGGTTAGAAAGGG
**mTAZ**	CGCCAGCAGTCCTATGACGTGAC	GCTGGGACACTGCCATGGACCTC
**mCTGF**	TGTGTACGGAGCGTGACCCCTGC	TTGCAGCTGCTTTGGAAGGACTC
**mKLF5**	ACTGCCCTCGGAGGAGCTGG	ATGCTCTGAAATTATCGGAACTG
**mINHBA**	AGGGACCCGAAAGAGAATTT	TAATCCAGCAACTTGCCAAC
**mSTYK1**	GGAGAGAAGGGTCACCTGAG	GCAAGAAGGATGAGAAAGCC
**mTHBS1**	CACCTCTCCGGGTTACTGAG	ATGCAACAGGAACAGGACAC
**mAngptl4**	GAATCTTCAGAGCCAGATAGAC	TTGGAAGAGTTCCTGGCAGTC
**mS100a7a**	CCTGCACCAAGAGCAACAGAC	GCAGCATAGTGATGGAAGCAG
**mAdamts5**	AGGTGTTTCCTTTGCTTGCT	CCGACCCACTTCCTTTCTTA
**hAldh1a1**	TGAGCCAGTCACCTGTGTTC	CCACTCACTGAATCATGCCA
**mAldh1a1**	CGGTGGATTCAAGATGTCTG	GATGTGTAGCCACTGCTGAGA
**mTnfrsf12a**	GCGCTGGTTTCTAGTTTCCT	AATGAATGAATGGACGACGA
**mrRNA**	AGGGGAGAGCGGGTAAGAGA	GGACAGGACTAGGCGGAACA
**mAldh1a2**	AGG GTC TCG CAT CTT TGT GG	TAT CGA TCT GAG GAC CCT GCT
**mAldh1a3**	CGG AGA GTG CGA ACC AGT TA	CCT TGT CCA CAT CGG GCT TA

Supplementary Table 4: Cellular genes upregulated by TAZ-S89A overexpression

Gene ID	Symbol	Fold increase	Function	Reference
11668	Aldh1a1	15.1	Cancer stem cells	Tomita16
21813	TgfbII	7.4	Cancer stem cell and metastasis	Bellomo 16
21825	Thbs1	6.6	Extracellular matrix protein, angiogenesis	Davis15
381493	S100a7a	6.6	Cell proliferation, apoptosis, immune response	Jia14
14219	Ctgf	6.3	Extracellular matrix protein, angiogenesis, wound healing	Krupska15
23794	Adamts5	5.3	Cell proliferation, cell invasion,	Kintakas11 Mochizuki S 07
16323	Inhba	4.8	Cell proliferation, cancer stem cells	Seder CW 09; Wamsley 15
12224	Klf5	3.7	Cell proliferation, cell survival, cancer stem cells	Farrugia 16
57875	Angptl4	3.3	Metastasis, metabolism, anoikis resistance	Tan12
243659	Styk1	3.2	EMT, metastasis,	Wang16

1. Tomita H, Tanaka K, Tanaka T, Hara A. Aldehyde dehydrogenase 1A1 in stem cells and cancer. Oncotarget. 2016; 7:11018–32. doi: 10.18632/oncotarget.6920.
2. Bellomo C, Caja L, Moustakas A. Transforming growth factor β as regulator of cancer stemness and metastasis. Br J Cancer. 2016; 115:761–9.
3. Davis PJ, Sudha T, Lin HY, Mousa SA. Thyroid Hormone, Hormone Analogs, and Angiogenesis. Compr Physiol. 2015; 6:353–62.
4. Jia J, Duan Q, Guo J, Zheng Y. Psoriasin, a multifunctional player in different diseases. Curr Protein Pept Sci. 2014; 15:836–42.
5. Krupska I, Bruford EA, Chaqour B. Eyeing the Cyr61/CTGF/NOV (CCN) group of genes in development and diseases: highlights of their structural likenesses and functional dissimilarities. Hum Genomics. 2015; 9:24.
6. Kintakas C, McCulloch DR. Emerging roles for ADAMTS5 during development and disease. Matrix Biol. 2011; 30:311–7.
7. Mochizuki S, Okada Y. ADAMs in cancer cell proliferation and progression. Cancer Sci. 2007; 98:621–8.
8. Seder CW, Hartojo W, Lin L, Silvers AL, Wang Z, Thomas DG, Giordano TJ, Chen G, Chang AC, Orringer MB, Beer DG. Upregulated INHBA expression may promote cell proliferation and is associated with poor survival in lung adenocarcinoma. Neoplasia. 2009; 11:388–96.
9. Wamsley JJ, Kumar M, Allison DF, Clift SH, Holzknecht CM, Szymura SJ, Hoang SA, Xu X, Moskaluk CA, Jones DR, Bekiranov S, Mayo MW. Activin upregulation by NF-κB is required to maintain mesenchymal features of cancer stem-like cells in non-small cell lung cancer. Cancer Res. 2015; 75:426–35.
10. Farrugia MK, Vanderbilt DB, Salkeni MA, Ruppert JM. Kruppel-like Pluripotency Factors as Modulators of Cancer Cell Therapeutic Responses. Cancer Res. 2016; 76:1677–82.
11. Tan MJ, Teo Z, Sng MK, Zhu P, Tan NS. Emerging roles of angiopoietin-like 4 in human cancer. Mol Cancer Res. 2012; 10:677–88.
12. Wang Z, Qu L, Deng B, Sun X, Wu S, Liao J, Fan J, Peng Z. STYK1 promotes epithelial-mesenchymal transition and tumor metastasis in human hepatocellular carcinoma through MEK/ERK and PI3K/AKT signaling. Sci Rep. 2016; 6:33205.
