# Supplementary material for: TAZ induces lung cancer stem cell properties and tumorigenesis by up-regulating ALDH1A1
Source: Oncotarget. 2017 Mar 21;8(24):38426–43. doi: 10.18632/oncotarget.16430 (PMC5503543; doi:10.18632/oncotarget.16430)
Supplement: Supplementary file 2 [file oncotarget-08-38426-s002.docx]

**Supplementary Table 1: Cellular genes upregulated by TAZ-S89A overexpression**

| **Gene ID** | **Symbol** | **log2 Ratio** |
| --- | --- | --- |
| 69169 | Faim3 | 6.92 |
| 71874 | 2310007B03Rik | 6.12 |
| 13113 | Cyp3a13 | 4.16 |
| 208098 | Panx3 | 4.10 |
| 14563 | Gdf5 | 4.00 |
| 229672 | Bcl2l15 | 3.93 |
| 381493 | S100a7a | 3.72 |
| 259144 | Olfr456 | 3.49 |
| 17540 | Mrvi1 | 3.49 |
| 14219 | Ctgf | 3.43 |
| 12224 | Klf5 | 3.30 |
| 59091 | Jph2 | 3.30 |
| 26358 | Aldh1a7 | 3.20 |
| 70701 | Nipal1 | 3.02 |
| 57875 | Angptl4 | 3.00 |
| 326623 | Tnfsf15 | 2.94 |
| 240873 | Tnfsf18 | 2.91 |
| 23928 | Lamc3 | 2.91 |
| 21825 | Thbs1 | 2.90 |
| 57425 | U90926 | 2.83 |
| 18158 | Nppb | 2.78 |
| 23794 | Adamts5 | 2.78 |
| 15945 | Cxcl10 | 2.72 |
| 210274 | Shank2 | 2.65 |
| 20392 | Sgce | 2.59 |
| 16323 | Inhba | 2.57 |
| 19260 | Ptpn22 | 2.49 |
| 76884 | Cyfip2 | 2.44 |
| 380863 | Tmem171 | 2.35 |
| 12722 | Clca1 | 2.34 |
| 12954 | Cryaa | 2.23 |
| 66985 | Rassf7 | 2.15 |
| 11668 | Aldh1a1 | 2.09 |
| 106565 | Dlk2 | 2.08 |
| 381232 | 5830416P10Rik | 2.07 |
| 14172 | Fgf18 | 2.05 |
| 20324 | Sdpr | 2.05 |
| 50768 | Dlc1 | 1.98 |
| 12365 | Casp14 | 1.98 |
| 217325 | Llgl2 | 1.95 |
| 23972 | Papss2 | 1.92 |
| 23882 | Gadd45g | 1.89 |
| 17873 | Gadd45b | 1.89 |
| 224796 | Clic5 | 1.87 |
| 13874 | Ereg | 1.86 |
| 68178 | Cgnl1 | 1.86 |
| 11443 | Chrnb1 | 1.83 |
| 108897 | Aif1l | 1.83 |
| 71026 | Speer3 | 1.77 |
| 13614 | Edn1 | 1.76 |
| 243659 | Styk1 | 1.73 |
| 14199 | Fhl1 | 1.73 |
| 11504 | Adamts1 | 1.73 |
| 242316 | Gdf6 | 1.70 |
| 76453 | Prss23 | 1.68 |
| 13835 | Epha1 | 1.68 |
| 14200 | Fhl2 | 1.68 |
| 30938 | Fgd3 | 1.68 |
| 20620 | Plk2 | 1.67 |
| 18787 | Serpine1 | 1.67 |
| 21679 | Tead4 | 1.66 |
| 110310 | Krt7 | 1.65 |
| 244550 | Podnl1 | 1.65 |
| 231440 | Parm1 | 1.65 |
| 18553 | Pcsk6 | 1.64 |
| 22793 | Zyx | 1.64 |
| 19153 | Prx | 1.62 |
| 16007 | Cyr61 | 1.60 |
| 83770 | Tas1r2 | 1.58 |
| 432530 | Adcy1 | 1.57 |
| 227731 | Slc25a25 | 1.57 |
| 21929 | Tnfaip3 | 1.56 |
| 56047 | Msln | 1.54 |
| 226251 | Ablim1 | 1.51 |
| 73683 | Atg16l2 | 1.51 |
| 237523 | Ptprq | 1.49 |
| 72789 | Veph1 | 1.49 |
| 100342 | Fam46b | 1.49 |
| 66234 | Sc4mol | 1.48 |
| 12832 | Col5a2 | 1.48 |
| 18793 | Plaur | 1.47 |
| 74769 | Pik3cb | 1.44 |
| 23917 | Impdh1 | 1.43 |
| 21813 | Tgfbr2 | 1.42 |
| 15982 | Ifrd1 | 1.40 |
| 16835 | Ldlr | 1.38 |
| 18442 | P2ry2 | 1.36 |
| 19288 | Ptx3 | 1.35 |
| 14573 | Gdnf | 1.35 |
| 242109 | Zfp697 | 1.35 |
| 227736 | 1700019L03Rik | 1.35 |
| 20296 | Ccl2 | 1.34 |
| 18049 | Ngf | 1.34 |
| 16449 | Jag1 | 1.34 |
| 68272 | Rbm28 | 1.33 |
| 20515 | Slc20a1 | 1.32 |
| 17760 | Mtap6 | 1.31 |
| 73086 | Rps6ka5 | 1.31 |
| 12286 | Cacna1a | 1.30 |
| 15200 | Hbegf | 1.30 |
| 52428 | Rhpn2 | 1.30 |
| 21885 | Tle1 | 1.29 |
| 192156 | Mvd | 1.28 |
| 109624 | Cald1 | 1.27 |
| 328162 | Trmt61a | 1.27 |
| 12457 | Ccrn4l | 1.26 |
| 50523 | Lats2 | 1.26 |
| 16367 | Irs1 | 1.26 |
| 69219 | Ddah1 | 1.25 |
| 11475 | Acta2 | 1.24 |
| 67896 | Ccdc80 | 1.24 |
| 24030 | Mrps12 | 1.23 |
| 627626 | 3110082D06Rik | 1.23 |
| 20563 | Slit2 | 1.23 |
| 20779 | Src | 1.22 |
| 14086 | Fscn1 | 1.21 |
| 17913 | Myo1c | 1.20 |
| 14066 | F3 | 1.19 |
| 208618 | Etl4 | 1.18 |
| 77994 | 2810055G20Rik | 1.18 |
| 243958 | Siglecg | 1.17 |
| 18591 | Pdgfb | 1.16 |
| 56772 | Mllt11 | 1.16 |
| 216795 | Wnt9a | 1.15 |
| 30059 | Timm10 | 1.15 |
| 20220 | Sap18 | 1.15 |
| 22403 | Wisp2 | 1.15 |
| 109711 | Actn1 | 1.14 |
| 260409 | Cdc42ep3 | 1.14 |
| 68659 | Fam198b | 1.14 |
| 68241 | Fam195a | 1.13 |
| 107094 | Rrp12 | 1.13 |
| 14725 | Lrp2 | 1.12 |
| 83397 | Akap12 | 1.12 |
| 18263 | Odc1 | 1.12 |
| 27279 | Tnfrsf12a | 1.11 |
| 16475 | Jub | 1.09 |
| 109229 | Fam118b | 1.09 |
| 70617 | 5730508B09Rik | 1.09 |
| 70737 | Cgn | 1.09 |
| 18759 | Prkci | 1.09 |
| 56332 | Amotl2 | 1.09 |
| 27966 | Rrp9 | 1.08 |
| 22779 | Ikzf2 | 1.07 |
| 56289 | Rassf1 | 1.07 |
| 12609 | Cebpd | 1.06 |
| 12977 | Csf1 | 1.06 |
| 235040 | Atg4d | 1.06 |
| 330171 | Kctd10 | 1.06 |
| 15417 | Hoxb9 | 1.06 |
| 74094 | Tjap1 | 1.06 |
| 56878 | Rbms1 | 1.05 |
| 94242 | Tinagl1 | 1.05 |
| 70350 | Basp1 | 1.05 |
| 67263 | Zswim6 | 1.05 |
| 20856 | Stc2 | 1.05 |
| 107765 | Ankrd1 | 1.05 |
| 319801 | 9630033F20Rik | 1.03 |
| 380928 | Lmo7 | 1.03 |
| 74127 | Krt80 | 1.03 |
| 12795 | Plk3 | 1.02 |
| 66143 | Eef1e1 | 1.02 |
| 18858 | Pmp22 | 1.02 |
| 320982 | Arl4c | 1.02 |
| 70788 | Klhl30 | 1.01 |
| 15229 | Foxd1 | 1.00 |
| 80915 | Dusp12 | 1.00 |
| 67876 | Coq10b | 1.00 |
